# Reliability, validity and clinical correlates of the Quality of Life in Alzheimer’s disease (QoL-AD) scale in medical inpatients

**DOI:** 10.1186/s12955-016-0493-8

**Published:** 2016-06-14

**Authors:** Gustav Torisson, Lars Stavenow, Lennart Minthon, Elisabet Londos

**Affiliations:** Clinical Memory Research Unit, Lund University, Simrisbanvägen 14, S 20502 Malmö, Sweden; Deparment of Internal Medicine, Skåne University Hospital, Malmö, Sweden

**Keywords:** Quality of life, Medical inpatients, Scale validity

## Abstract

**Background:**

There is a lack of standardisation in quality of life (QoL) measurements to be used in older multimorbid patients. An ideal QoL measurement should be reliable, valid, subjective, multidimensional, feasible and generic. We hypothesised that the QoL-AD (Quality of Life in Alzheimer’s Disease) scale could have these properties. Our aim was to determine the psychometric properties and clinical correlations of QoL-AD in a population of elderly, multimorbid medical inpatients.

**Methods:**

QoL-AD was performed in 200 medical inpatients, and available caregivers. Reliability was determined using cronbach’s alpha and corrected item-total correlations. The agreement between patient and proxy ratings were examined using intra-class correlations (ICC). Correlations between QoL-AD and demographic data, comorbidity, cognitive tests, ADL (activities of daily living) and depression were examined. To characterise the underlying constructs of QoL-AD, an exploratory factor analysis was performed.

**Results:**

In total, 199 patients fulfilled the QoL-AD rating, with 139 proxy ratings. Cronbach’s alpha (95 % CI) was 0.74 (0.68–0.79) for patients and 0.86 (0.83–0.90) for proxies. Patient-proxy ICC (95 % CI) was 0.31 (0.16–0.46). Lower QoL was correlated to depression, cognitive impairment, ADL impairment and solitary living, but not with comorbidity. The factor analysis gave a three-factor solution, with factors representing phsyical, social and psychological well-being.

**Conclusion:**

The QoL-AD scale showed some promising properties but more research is needed before it can be recommended in this setting. If replicated, the finding that cognitive impairment, depression and ADL impairment were more associated with lower QoL than somatic comorbidity could have clinical implications for further studies aiming to improve QoL in this population.

**Electronic supplementary material:**

The online version of this article (doi:10.1186/s12955-016-0493-8) contains supplementary material, which is available to authorized users.

## Background

Quality of life (QoL) has been described by the World Health Organisation as “a broad ranging concept affected in a complex way by the person’s physical health, psychological state, level of independence, social relationships, personal beliefs and their relationship to salient features of their environment [1]”.

Multimorbidity, defined as having two or more chronic conditions is strongly associated with lower QoL [2–4]. A recent study showed that more than 60 % of primary care patients aged over 65 years were multimorbid [5]. The association between QoL and multimorbidity is well established in community settings [2–4, 6–8]. Studies in hospital settings are much more scarce but suggest that multimorbidity could pose an even larger impact on QoL [9, 10].

Lack of standardisation in QoL measures in older age is a concern [11]. An ideal QoL instrument in elderly should have been developed in an older population and include subjectivity and multidimensionality. It should be generic rather than disease-specific and at the same time brief and feasible [11].

The Quality of Life in Alzheimer’s Disease (QoL-AD) scale fulfils several of these criteria but was originally developed as a disease-specific scale for Alzheimer’s disease [12]. Lately, the QoL-AD has been applied in more diverse populations, such as in residential homes, in non-demented elderly and in patients with Lewy-Body disease [12–16]. Across settings, previous studies have shown good to excellent reliability of the QoL-AD [13, 16–22]. Typically, patients rate their QoL higher than their caregivers [12, 13, 16–23]. Lower QoL-AD results have been associated with depression, cognitive impairment, impaired ADL and higher comorbidity to a various degree [12, 17, 19, 23, 24]. A summary of previous studies on QoL-AD is found in Table 1.

**Table 1:** Previous studies of QoL-AD

Author	Setting	n	age (mean)	female sex	MMSE (mean)	Cronbach alpha		QoL-AD mean score		Corr.	Item-total corr. (range)		Correlated measures
						pat.	car.	pat.	car.	pat -car.	pat.	car.	
Bosboom [24]	community	80	78	65 %	>10	-	-	32	30	-	-	-	Depression, cognition
Condé - Sala [23]	outpatients with AD	236	78	67 %	-	-	-	34	31				Living alone, sex, depression, ADL, NPI
Buasi [13]	mild to moderate AD	136	76	67 %	17	.82	.82	-	-	-	.40–.68^e^	.35–71	-
Logsdon [12]	AD patients	77	78	47 %	17	.88	.87	38	33	.40^c^	.41–.67^e^	.34–.60	Depression, cognition, ADL
Thorgrimsen [16]	dementia	201	85	79 %	14	.82	-	33	-		>0.3^e^	-	Depression, EQ-5D^a^, D-QoL^b^
Matsui [20]	mild to moderate AD	140	72	60 %	20	.84	.82	29	25	.60^c^	.18–.67^e^	.12–.55	Cognition, mood, age, NPI
Novelli [21]	mild to moderate AD	60	76	70 %	-	.80	.86	36	31	.35^c^	.27–.70^f^	.43–.68	Depression, NPI, cognition, ADL, WHOQoL^a^
Barrios [17]	MCI or mild-moderate dementia	104	77	68 %	21	.87	.86	29	25	.26^d^	.35–.73^g^	.36–.69	ADL, depression, cognition, NPI, comorbidity
Leon-Salas [18]	nursing home	101	83	88 %	12	.86	.90	34	31	-	.28–.84^g^	.11–.67	Depression, ADL, NPI, Eq-VAS^a^, Qualid^b^
Wolak [22]	mild to moderate AD	120	82	64 %	21	.83	.79	36	33	.43^d^			Duke health profile^a^, NPI
Logsdon [19]	probable or possible AD	177	77	44 %	18	.84	.86	-	33	.28^d^	-	-	ADL, depression
Torisson (this study)	medical inpatients	200	83	65 %	23	.74	.86	33	31	.31^d^	.13–.56^g^	.31–.66	ADL, depression, living alone, cognition

Previous studies on the properties of the QoL-AD scale

*AD* Alzheimer’s disease, *MCI* mild cognitive impairment, *pat.* patient rating, *car.* caregiver rating, *MMSE* mini-mental state examination, *QoL-AD* Quality of Life in Alzheimer’s disease, *ADL* activities of daily living, *NPI* neuropsychiatric inventory

^a^generic QoL instrument

^b^dementia-specific QoL instrument

^c^Pearson correlation used

^d^Intraclass-correlation was used

^e^not specified which correlation used

^f^spearman correlation used

^g^corrected item-total correlation used

In a previous study, we found that undetected cognitive impairment was frequent in medical inpatients [25]. We hypothesised that the QoL-AD could be a suitable measurement in this setting. In the present study, we aim to determine the clinical correlations, reliability and validity of QoL-AD in this elderly, multimorbid hospital population.

## Methods

The current study constitutes a secondary analysis, patients were concurrently participating in a previously published prospective intervention study [26].

### Setting

The study was carried out at the department of General Internal Medicine at Skåne University Hospital in Malmö, the third largest city in Sweden, with approximately 300.000 citizens. The hospital is the only inpatient facility in the city, providing tertiary care to its inhabitants. Patients at the wards of the department of General Internal Medicine are primarily elderly with multiple conditions. The majority of patients are admitted through the hospital’s emergency department, with a wide variety of presenting complaints. Many patients receive community care at home and undergo discharge planning before returning home or to an institutional living.

### Patients

Admitted patients over 60 years of age and living in a non-institutional living were considered eligible. Exclusion criteria included hospital-associated criteria (transfer to another department/intensive care, discharge before inclusion, isolation due to contagious disease). Patients had to be able to perform cognitive and functional tests, therefore patients with language barrier, terminal disease, blindness, deafness, aphasia or altered conciousness were excluded as well. In all, two hundred patients were included, the study inclusion has been described in detail before, including a detailed flowchart [26].

Included patients were taking part in a concurrent intervention study aiming to increase quality of care. Interventions included a comprehensive medication overview, liaison with GP at discharge, improved discharge planning and post-discharge telephone follow-up. Of the 200 patients, 99 patients received the interventions and 101 patients standard care. Group allocation (control/intervention) was carried out using convenience sampling through geographic selection, i.e. the study was not randomised.

### Baseline measurements

The patients underwent a baseline measurement consisting of an interview (with a caregiver if available), medical record review, cognitive tests, functional tests and the QoL measurment**.**

To measure comorbidity, the Charlson comorbidity index was used [27]. As an alternative measure, the total number of drugs was noted (drugs taken “as needed” were not included). This data were retreived from interviews first and then completed with data from electronic medical records.

Cognitive impairment was measured with the mini-mental state examination (MMSE) and the clock-drawing test (CDT) [28, 29]. Both tests were carried out during the hospitalisation, in a calm environment at the ward, when the patients were stabilised. MMSE ranges from from 0 (worst) to 30 (best). The CDT was rated using the six-point scale of Shulmann, ranging from 0 (worst) to 5 (best) [29].

The Gottfries-Bråne-Steen, or GBS, scale was also employed [30, 31]. This scale is comprised of four subsets: intellectual functions, emotional functions, ADL functions (activities of daily living) and symptoms. For the current study, the GBS-ADL subset and the “depressed mood” symptom was considered. The GBS-ADL subset is performance-based and comprised of six items (dressing, food intake, physical activity, spontaneous activity, continence and toileting). These are rated from 0 (best) to 6 (worst), for a total score of 0–36. As a proxy for ADL impairment we included the anamnestic data regarding access to community home care (yes/no). The GBS “depressed mood” symptom was also rated from 0 (best) to 6 (worst). As an alternative estimate of depression, we noted if patients were taking antidepressants (yes/no). Any drug in group N06A in the ATC (Anathomical Therapeutic Chemical) classification system was considered an antidepressant. Thus, two measures were collected each regarding physical comorbidity, cognitive impairment, ADL impairment and depression.

### Quality of Life in Alzheimer’s disease scale (QoL-AD)

The QoL-AD scale was developed by Logsdon et al. [12, 19]. The QoL-AD is comprised of 13 items (physical health, energy, mood, living situation, memory, family, marriage, friends, self as a whole, ability to do chores, ability to do things for fun, money and life as a whole). Response options include 1(poor), 2(fair), 3(good) and 4 (excellent), for a total score of 13–52, with higher scores indicating better QoL.

The patients’ ratings were performed in an interview, with standardised instructions to avoid influencing the results. Interviews were done in a calm environment at the hospital ward by a team of two occupational therapists and a nurse, all of whom had received special training prior to the study. Caregiver ratings were done separately, using a questionnaire.

The patient and caregiver ratings were combined into a weighted composite score in the same way as in the original paper by Logsdon et al.: (2 × patient score + 1 × caregiver score)/3 [12].

Several studies have used factor analysis to describe underlying constructs in the QoL-AD, with diverse results [13–16, 22]. The most comprehensive of these studies, that perform exploratory and confirmatory factor analysis in a large sample of non-demented elderly, has reached a three-factor solution, with factors representing physical, social and psychological domains of quality of life [15].

### Statistical analysis

For QoL-AD, we used the strategy suggested in the original paper on missing values [12]. If a case had one or two missing items, they were imputed with that case’s mean value. If more than two items were missing, the case was discarded.

Internal consistency of QoL-AD was determined using Cronbach’s alpha, where a value of >0.7 is generally considered desirable. In addition, corrected item-total correlations between the 13 separate items and the total score were determined. Ideally, these should reach > 0.3 for all items. The conformity of patient and caregiver ratings was determined using a two-way mixed model single-measure intra-class correlation coefficient (ICC). The mean difference between the total score of patient and caregiver ratings was tested using a paired-sample t test.

Clinical associations were determined by using the Spearman’s correlations with the other baseline measurements, for each of the 13 QoL-AD items separately and with the Pearson correlation for the total score. A crude Bonferroni correction for multiple comparisons was utilised (all p values were multiplied with the number of variables, 12. Thus a p value of 0.004 was needed for a correlation to be considered significant). Control/intervention allocation in the concurrent intervention study was included as well, to detect selection bias.

Construct validity was also examined using an exploratory factor analysis on the QoL-AD items. Factorability was determined using Bartlett’s test of sphericity and the Kaiser-Mayer-Olkin test, where the latter should ideally exceed 0.5. Extraction was done on the correlation matrix, using principal factor analysis. Factors with Eigenvalues > 1 were retained. Factors were rotated using an orthogonal Varimax rotation [32, 33].

All analyses were carried out using SPSS version 20.0.

## Results

Of the 200 patients, 199 finished the QoL-AD rating. A total of four datapoints were missing (one each on “marriage”, “living situation”, “ability to do chores” and “life as a whole”) and were imputed accordingly. The caregivers completed 141 ratings. Of these, two ratings were missing more than two items and were discarded. In the remaining 139, eight datapoints were missing (two “family” and six “marriage”) and were imputed. No other imputations were done.

The mean age was 83.4 years and 65 % of patients were female. Regarding living arrangements, 59 % had home care and 67 % were living alone. There were no significant differences between the full sample and the sample with a caregiver rating, the baseline characteristics for the two groups are shown in Table 2.

**Table 2:** Baseline characteristics

Variable	Full sample *n* = 199	Subset with caregiver rating *n* = 139
Age	83.4 (8.1)	83.7 (7.4)
Female sex	130 (65 %)	94 (68 %)
Living alone	134 (67 %)	90 (65 %)
Comorbidity - Charlson comorbidity index	2.3 (1.5)	2.1 (1.5)
Comorbidity - Number of drugs	7.1 (3.9)	7.0 (3.9)
Cognition - Mini-mental state examination	22.9 (4.2)	22.6 (4.5)
Cognition - Clock-drawing test	3.4 (1.2)	3.3 (1.2)
Function - GBS - ADL	6.8 (5.7)	6.9 (5.8)
Function - Home care	118 (59 %)	79 (57 %)
Depression - GBS - depression	0.9 (1.0)	0.8 (0.9)
Depression - On antidepressants	31 (16 %)	21 (15 %)
Intervention in original study	99 (50 %)	60 (43 %)

Characteristics of the full sample and the subsample with caregiver ratings. Data is presented as mean (standard deviation) or number (percentage)

*GBS* Gottfries-Bråne-Steen scale, *ADL* activities of daily living, *QoL-AD* Quality of Life in Alzheimer’s disease scale

Caregivers were generally scoring the QoL-AD lower than patients, which also was reflected in the total score (mean 33.3 vs 30.6, pairwise t-test = 4.36, *p* < 0.001). All items except money/economic situation were rated lower by the caregivers than the patients. The intra-class correlation coefficients between patient and caregiver ratings for the separate items ranged from 0.05 (“physical health” item) to 0.55 (“marriage “item). For the total score, the ICC was 0.31 (95 % CI 0.16–0.46). Patient and caregiver ratings, composite score and intra-class correlations for the separate items are shown in Table 3.

**Table 3:** Patient and caregiver scores on QoL-AD

QoL-AD item	Patient score mean (SD)	Caregiver score mean (SD)	Composite score mean (SD)	ICC patient - caregiver
Physical	2.0 (0.8)	1.7 (0.7)	1.9 (0.6)	0.05
Energy	1.9 (0.8)	1.8 (0.8)	1.9 (0.7)	0.23**
Mood	2.5 (0.8)	2.2 (0.8)	2.4 (0.6)	0.25**
Living situation	3.3 (0.7)	3.0 (0.9)	3.2 (0.6)	0.25**
Memory	2.4 (0.8)	2.4 (1.0)	2.4 (0.7)	0.35***
Family	3.4 (0.7)	3.1 (0.9)	3.3 (0.6)	0.34***
Marriage	2.8 (0.9)	2.6 (1.0)	2.8 (0.8)	0.52***
Friends	2.7 (0.9)	2.4 (1.0)	2.6 (0.8)	0.29***
Self as a whole	2.4 (0.8)	2.3 (0.7)	2.3 (0.6)	0.13
Ability to do chores	2.1 (0.8)	1.9 (0.8)	2.0 (0.7)	0.28***
Ability to do things for fun	2.3 (0.9)	2.0 (1.0)	2.2 (0.8)	0.27***
Money	2.8 (0.8)	2.9 (0.8)	2.8 (0.6)	0.38***
Life as a whole	2.8 (0.8)	2.4 (0.9)	2.6 (0.6)	0.15*
Total score	33.3 (5.2)	30.6 (7.1)	32.4 (4.8)	0.31***

Patient and caregiver ratings

*ICC* intraclass correlation coefficient

*p <0.05

**p < 0.01

***p < 0.001

The internal consistency, measured by Cronbach’s alpha, for patients’ rating was 0.74 (95 % CI 0.68–0.79). The caregiver ratings and the composite scores were higher (0.86 and 0.80, respectively). The corrected item-total correlations ranged from 0.13 (“memory” item) to 0.56 (“ability to do things for fun” item) for the patients and from 0.31 (“money” item) to 0.66 (“life as a whole” item) for the caregivers. The separate corrected item-total correlations are presented in Table 4.

**Table 4:** Internal consistency

QoL-AD item	Item-total correlations patient	Item-total correlations caregiver	Item-total correlations composite
Physical	0.31	0.45	0.33
Energy	0.38	0.51	0.35
Mood	0.47	0.58	0.54
Living situation	0.28	0.54	0.51
Memory	0.13	0.52	0.27
Family	0.36	0.35	0.33
Marriage	0.34	0.58	0.41
Friends	0.33	0.62	0.44
Self as a whole	0.40	0.62	0.45
Ability to do chores	0.49	0.52	0.53
Ability to do things for fun	0.56	0.63	0.64
Money	0.27	0.31	0.24
Life as a whole	0.37	0.66	0.53
Cronbach’s alpha	0.742	0.863	0.797

Corrected item-total correlations for all items and Cronbach’s alpha, for patient ratings, caregiver ratings and the composite score

Regarding clinical associations, all significant correlations had the expected direction. The clinical correlations of caregiver ratings were generally stronger than those of the patients ratings. In the patients’ ratings, lower QoL-AD scores were correlated with depression, functional impairment and solitary living. The caregiver QoL-AD ratings had the same correlations, but with the addition of cognitive impairment, see Table 5. All the separate QoL-AD items’ correlations for the composite scores are presented in Additional file 1: Table S1.

**Table 5:** Clinical correlations

Variable	Expected	Patient	Caregiver	Composite
Age	-	−.08	−.21	−.16
Female sex	-	−.14	−.14	−.22
Charlson index	-	−.05	−.11	−.11
No. of drugs	-	−.11	−.10	−.16
GBS - mood	-	−.26^a^	−.24^a^	−.28^a^
Antidepressant use	-	−.10	−.26^a^	−.23
MMSE	+	.17	.41^a^	.35^a^
CDT	+	.06	.36^a^	.31^a^
GBS-ADL	-	−.22^a^	−.42^a^	−.38^a^
Home care	-	−.26^a^	−.39^a^	−.40^a^
Living alone	-	−.24^a^	−.35^a^	−.32^a^
Group in original study	none	.03	.01	.01

Correlation coefficients between QoL-AD total scores and the other measurements. “Group in original study” denotes group allocation in the original intervention study, to detect selection bias. “expected” denotes the a priori hypothesised direction of correlation

*GBS* Gottfries-Bråne-Steen scale, *MMSE* mini-mental state examination, *CDT* clock-drawing test, *ADL* activities of daily living

^a^significant correlation after Bonferroni correction

For the factor analysis, no violation was found regarding the underlying assumptions regarding factorability of the results; the Kaiser-Meyer-Olkin test result was 0.82. The Bartlett test of Sphericity was highly significant (χ^2^ = 430, d.f. = 78, *p* < 0.001). Three factors had unrotated eigenvalues > 1, with 3.94, 1.73 and 1.23, respectively. The fourth component, which was not retained, had a value of 0.96. The three factor solution explained 40 % of the variance. The three were labelled factors Social (comprised of “living situation”, “family”, “marriage”, “friends”, “money”, “life as a whole”), Physical (“physical health”, “energy”, “ability to do chores”, “ability to do things for fun”) and Psychological (“mood”, “memory”, “self as a whole”). The communalities, post-rotation loadings and percentage variation are shown in Table 6.

**Table 6:** Factor analysis

QoL-AD item	Factor 1: physical	Factor 2: social	Factor 3: psychological	h^2^
Energy	0.64			
Ability to do things for fun	0.63			
Physical	0.60			
Ability to do chores	0.59			
Marriage		0.67		
Living situation		0.61		
Friends		0.51		
Family		0.42		
Money		0.36		
Life as a whole		0.44		
Self as a whole			0.64	
Mood			0.55	
Memory			0.51	
% variance	16 %	15 %	9 %	40 %

Exploratory factor analysis of the composite score. The rotated factor solution is displayed. Percentage variance is post-rotation. Factor loading values below .35 are not included h^2^ = communalities

## Discussion

In this study, we employed the QoL-AD for the first time in a medical hospital population. Analysis of reliability and validity were largely similar to previous studies, suggesting that QoL-AD may be suitable for this population. Regarding clinical associations, we found that lower QoL was associated with depression, cognitive impairment, ADL impairment and solitary living.

In a recent comprehensive study, using data from two clinical trials of Bapinezeumab in patients with mild to moderate Alzheimer’s disease, Lacey and colleagues evaluated the utility of the QoL-AD scale [34]. The authors found that patient-rated QoL-AD was lower than caregiver-rated, consistent with all previous QoL_AD studies. Furthermore, the authors found that QoL-AD was only weakly associated with clinical measures of cognition and function, with caregiver-rated QoL-AD having a slightly stronger association with clinical measures. The authors concluded that QoL-AD was not suited to measure disease progression.

Albeit in a different setting, this pattern is partly reoccuring in our study, patients rated their QoL higher than their caregivers. The intra-class correlation between patient and proxy ratings were modest with an ICC of 0.31, in line with previous studies [19, 22, 35]. Also, regarding clinical associations, there was a discrepancy with caregiver ratings having stronger correlations with clinical measures than patients’ ratings. Specifically, this concerned cognitive tests, with no correlation for the self-reported score, a finding shown in several other studies [35–37].

Lacey et al. suggest that this pattern could be attributed to lacking insight as a consequence of cognitive impairment [34]. However, other studies have not been able to prove an effect of lacking insight on QoL ratings in milder forms of dementia [35, 37]. Our population was not demented but rather multimorbid. Another possible explanation is adaptation, or what is known as “response shift”, a gradual adjustment to chronic disease that has been shown in other diseases as well [35]. According to this hypothesis, patients adapt to a lower level of function, while caregivers retain a former, higher, level as their benchmark [34].

Concerning physical comorbidity, neither the highly established Charlson index nor number of drugs were associated with QoL-AD score. This is an interesting finding, as our study is the first using QoL-AD in a somatic hospital setting. One possible explanation is that the QoL-AD does not measure the effect of physical health on QoL in the expected way and thus is not a valid QoL measurement for this population. Another possibility is that these patients, and their caregivers, actually consider mental health and cognitive impairment to have a much larger impact on QoL than physical comorbidity.

In the factor analysis, we found a three-factor solution, containing the factors “physical well-being”, “social well-being” and “psychological well-being”. This structure with three different constructs is the exact same that was found in non-demented community-dwelling elderly in a high-quality previous study [15]. According to the WHO definition, QoL is a multi-dimensional concept and the three-factor solution with physical, mental and social domains is similar to many other theoretical constructs, including for example the Neuro-QoL initiative [38].

An ideal QoL instrument in this setting should be subjective, multidimensional, generic and feasible. The only prerequisite not fulfilled by QoL-AD is that it was not developed as a generic measure but a disease-specific measure for Alzheimer’s disease. However, only one of 13 items directly concerns cognitive impairment (the memory item). Interestingly, a new scale, the WHOQOL-AGE, has been developed as a generic instrument for use in the elderly [39]. The WHOQOL-AGE is very similar to the QoL-AD, it contains 13 items rated from 1 to 5, of which 9 have direct counterparts in the QoL-AD. This similarity between these two scales supports the impression that QoL-AD may have more generic properties than originally thought.

However, the WHOQOL-AGE does not include cognitive impairment. Whether cognitive impairment should be seen as a core aspect of QoL or a predictor of QoL could be debated. In our material, 73 % had cognitive impairment, of which the majority were undetected previously. Furthermore, the memory item by itself had a correlation with MMSE of 0.45 for the composite score, see Additional file 1: Table S1. Thus, in our opinion, cognitive impairment should be a part of the QoL assessment in this setting.

Looking ahead, there are several other state-of-the-art initiatives , such as PROMIS (patient reported outcomes measurement information system) (www.nihpromis.org) and NeuroQoL that could possibly adress the multimorbidity issue [38]. These systems apply item response theory (IRT) and computer adaptive testing (CAT) to tailor a test, where the reply on one item is used to select the next one from a large item bank. This results in an individual score that is comparable across a range of conditions. The concept of adaptive individual testing in multimorbid elderly patients is very appealing, as it could combine generic and disease-specific properties. At the same time, with the right algorithm, the test could be broad-ranging, brief and precise.

Until such instruments are implemented generally, the lack of standardisation is a concern. In a review including 37 studies on QoL in older patients, a total of 28 different QoL measures were used, most of them developed for younger populations [11]. Only 3 generic QoL scales addressed cognitive impairment, the briefest one including 68 items.

## Conclusion

The QoL-AD showed some promising properties but it is to early to recommend QoL-AD in this setting without further research. A prospective study could determine the feasibility and validity of QoL-AD and compare it to other relevant QoL measures. A larger study could perform multivariate analysis to further analyse clinical correlates, controlling for the other parameters and address multicolinearity.

In the present study, cognitive impairment, ADL impairment and depression were more strongly associated with lower QoL than physical comorbidity. This could have important clinical implications. Today, physical health and social aspects are routinely adressed in medical inpatients. The same does not apply to cognitive impairment and depression which are often undetected, despite being associated with adverse outcomes [25, 40, 41]. If our findings were to be replicated, it would be yet another reason to increase acknowledgement of these issues in order to improve quality of life.

## Abbreviations

ADL, activities of daily living; CDT, clock-drawing test; CI, confidence interval; GBS, Gottfries-Bråne-Steen scale; ICC, intra-class correlation; MMSE, mini-mental state examination; QoL, quality of life; QoL-AD, Quality of Life in Alzheimer’s disease

## Additional file

Additional file 1: Convergent validity - composite rating. (DOC 74 kb)
