# Supplementary material for: Reliability, validity and clinical correlates of the Quality of Life in Alzheimer’s disease (QoL-AD) scale in medical inpatients
Source: Health Qual Life Outcomes. 2016 Jun 14;14:90. doi: 10.1186/s12955-016-0493-8 (PMC4908755; doi:10.1186/s12955-016-0493-8)
Supplement: Additional file 1: — Convergent validity - composite rating. (DOC 74 kb) [file 12955_2016_493_MOESM1_ESM.doc]

**Supplementary table - Convergent validity - composite rating**

| QoL-AD items | age | female sex | Charlson index | no. of drugs | GBS-mood | antidepressant use | MMSE | CDT | GBS-ADL | Home care | living alone | group in original study |
| --- | --- | --- | --- | --- | --- | --- | --- | --- | --- | --- | --- | --- |
| expected correlation | - | - | - | - | - | - | + | + | - | - | - | none |
| physical | .07 | .00 | -.15 | -.25* | -.12 | -.02 | .07 | .08 | -.05 | -.19 | .08 | -.09 |
| energy | .02 | -.05 | -.13 | -.13 | -.17 | -.11 | -.01 | .07 | -.10 | -.16 | .06 | -.08 |
| mood | .14 | -.18 | -.11 | -.10 | -.30* | -.21 | .20 | .21 | -.23 | -.10 | -.12 | .02 |
| living situation | -.20 | -.06 | -.04 | -.10 | -.13 | -.07 | .26 | .16 | -.29* | -.27* | -.35* | .06 |
| memory | -.07 | -.07 | .06 | .01 | -.19 | -.19 | .45* | .30* | -.33* | -.09 | -.11 | -.05 |
| family | -.03 | -.16 | -.05 | -.06 | -.12 | -.13 | .17 | .23 | -.18 | -.16 | -.12 | -.02 |
| marriage | -.16 | -.24* | .07 | .02 | -.08 | -.05 | .24 | .21 | -.25* | -.37* | -.57* | .00 |
| friends | -.24* | -.14 | -.05 | -.05 | -.16 | -.14 | .23 | .10 | -.24* | -.29* | -.15 | -.07 |
| self as a whole | -.03 | -.15 | -.13 | -.11 | -.12 | -.09 | .15 | .21 | -.05 | -.01 | -.04 | -01 |
| ability - chores | -.20 | -.02 | -.08 | -.18* | -.16 | -.13 | .19 | .23 | -.29* | -.42* | -.21 | .10 |
| ability - fun | -.19 | -.03 | -.10 | -.12 | -.33* | -.15 | .27* | .32* | -.32* | -.28* | -.13 | .04 |
| money | -.05 | -.27* | -.06 | -.04 | .08 | -.17 | .11 | -.04 | -.03 | -.17 | -.24 | .11 |
| life as a whole | -.05 | -.20 | -.08 | -.04 | -.16 | -.16 | .21 | .13 | -.20 | -.22 | -.27* | .04 |
| total score | -.16 | -.22 | -.11 | -.16 | -.28* | -.23 | .35* | .31* | -.38* | -.40* | -.32* | .01 |

table S1 Correlations between the separate QoL-AD items (composite rating) and other measurements. GBS = Gottfries-Bråne-Steen scale, MMSE = mini-mental state examination, CDT = clock-drawing test, ADL = activities of daily living. * = significant correlation after Bonferroni correction
